# APLP2 Modulates JNK-Dependent Cell Migration in* Drosophila*

**DOI:** 10.1155/2018/7469714

**Published:** 2018-07-29

**Authors:** Xingjun Wang, Xiaowei Guo, Yeqing Ma, Chenxi Wu, Wenzhe Li, Lei Xue

**Affiliations:** ^1^Department of Interventional Radiology, Shanghai 10th People's Hospital, Shanghai Key Laboratory of Signaling and Diseases Research, School of Life Science and Technology, Tongji University, Shanghai 200092, China; ^2^Department of Neuroscience, Scripps Research Institute Florida, 130 Scripps Way, Jupiter, Florida 33458, USA; ^3^College of Chinese Medicine, North China University of Science and Technology, 21 Bohai Road, Tangshan 063210, China

## Abstract

Amyloid precursor-like protein 2 (APLP2) belongs to the APP family and is widely expressed in human cells. Though previous studies have suggested a role of APLP2 in cancer progression, the exact role of APLP2 in cell migration remains elusive. Here in this report, we show that ectopic expression of APLP2 in* Drosophila* induces cell migration which is mediated by JNK signaling, as loss of JNK suppresses while gain of JNK enhances such phenotype. APLP2 is able to activate JNK signaling by phosphorylation of JNK, which triggers the expression of matrix metalloproteinase MMP1 required for basement membranes degradation to promote cell migration. The data presented here unraveled an* in vivo* role of APLP2 in JNK-mediated cell migration.

## 1. Introduction

Amyloid precursor-like protein-2 (APLP2) belongs to the protein family that includes amyloid precursor protein (APP) and amyloid precursor-like protein-1 (APLP1) in mammals [1, 2]. The three proteins show sequence similarity in the extracellular E1, E2 and the intracellular domains, while only APP and APLP2 share a special Kunitz protease inhibitor (KPI) domain and an Asp-Glu-rich domain, suggesting a role that is likely specific for the two proteins [3, 4]. Consistent with this notion, APP and APLP2 are widely expressed in many tissues, whereas APLP1 is predominantly restricted to the neural cells [5–7]. Studies in knockout mice have unraveled that all the single knockout and the APP/APLP1 double knockout mice are viable and fertile displaying no evident phenotype, while the double knockout mice of APP/APLP2 or APLP1/APLP2 are prenatally lethal, implying a specific role of APLP2 in animal development [8–10], which is consistent with their divergent protein interaction networks observed in an* in vivo* brain study [11]. Furthermore, the phylogenetic tree of APP protein family indicates that APLP2 is more distant from an inferred ancestral gene than APP and APLP1 [12], suggesting APLP2 may perform distinct* in vivo* functions.

APLP2 has been shown to regulate multiple cellular functions such as neurite outgrowth, axogenesis, corneal epithelial wound healing, cell adhesion, migration [13], and mitosis [14–17]. The expression level of APLP2 is upregulated in the pancreatic tumor cell lines S2-013, the prostate cancer cell line DU145, and certain human cancers such as breast cancer [18–20] but is downregulated in the lymphoma cell lines [21] and in the lung neuroendocrine tumors [22]. Thus, the exact role of APLP2 in tumorigenesis remains elusive.

The c-Jun N-terminal Kinase (JNK) pathway is a highly conserved signaling from* Drosophila* to human that governs diverse cellular functions including cell proliferation, differentiation, death, and migration and regulates physiological processes such as stress response and lifespan [23–28]. However, an* in vivo* role of APLP2 in modulating JNK signaling has not been characterized.

In this work, we studied the* in vivo *function of human APLP2 in* Drosophila*. We found that ectopic expression of APLP2 in the wing disc of 3^rd^ instar larvae promotes cell migration, which is suppressed by loss of JNK signaling while exacerbated by gain of JNK signaling. Consistently, APLP2 activates JNK signaling by the phosphorylation of JNK and thus elevates JNK target gene MMP1 expression to initiate cell migration. This work, therefore, provides the first* in vivo* function of APLP2 in JNK-mediated cell migration.

## 2. Materials and Methods

### 2.1. Fly Stocks

All the fly stocks were raised on standard* Drosophila* corn media and crosses were performed at 25°C unless otherwise indicated.* UAS*-APLP2 was kindly provided by Dr. Merders; *puc*^H246^,* UAS-puc*-IR,* UAS*-*mmp1*-IR, and* UAS-p*35 were obtained from Bloomington Stock Center;* UAS*-Bsk^DN^,* UAS*-puc[26],* ptc*-Gal4,* en*-Gal4, *puc*^E69^[29], and* UAS*-*LacZ* [30] were previously described.

### 2.2. Statistical Analysis for Cell Migration

More than 20 wing discs were dissected for each genotype. The number of migrating cells in the posterior compartment of the wing discs was counted. Unpaired* t* test by GraphPad Prism 5 was used to analyze the statistical significance. Error bars mean ± SEM, *∗∗∗*: p<0.001, *∗∗*: p<0.01, and n.s.: no significant difference.

### 2.3. X-Gal Staining

3^rd^ instar larvae wing discs were dissected in PBST and stained for *β* galactosidase activity[31]. The steps are shown below: dissect the 3^rd^ instar larvae in buffer A (50mL PBST +50ul 1mM MgCl_2_ +1.5mL 5M NaCl) (PBST +150mMNaCl+1mM MgCl_2_); fix the tissue in buffer A containing 1% glutaraldehyde for 15 minutes at 4°C; rinse the tissue once in buffer A containing 3.3 mM K_3_Fe(CN)_6_ and 3.3 mM K_4_Fe(CN)_6_.3H_2_O; incubate the tissue in buffer A containing 3.3 mM K_3_Fe(CN)_6_ and 3.3 mM K_4_Fe(CN)_6_.3H_2_O. and 0.2%5-bromo-4-chloro-3indolyl-*β*-D-galactopyranoside (X-gal) at RT 1-4h; and store the tissues in 100% glycerol at 4°C.

### 2.4. Immunohistochemistry

Antibody staining of the imaginal discs was performed as previously described [32]. Antibodies used are as follows: mouse anti-*β*-gal (1:400, Developmental Studies Hybridoma Bank), mouse anti-MMP1 (1:100, Developmental Studies Hybridoma Bank), and rabbit anti-phospho-JNK (1:200, Calbiochem). Secondary antibodies were anti-rabbit-Alexa (1:1000, Cell Signaling and Technology) and anti-mouse-Cy3 (1:1000, Jackson ImmunoResearch).

## 3. Results and Discussion

### 3.1. APLP2 Promotes Cell Migration in* Drosophila*

**Figure 1:**
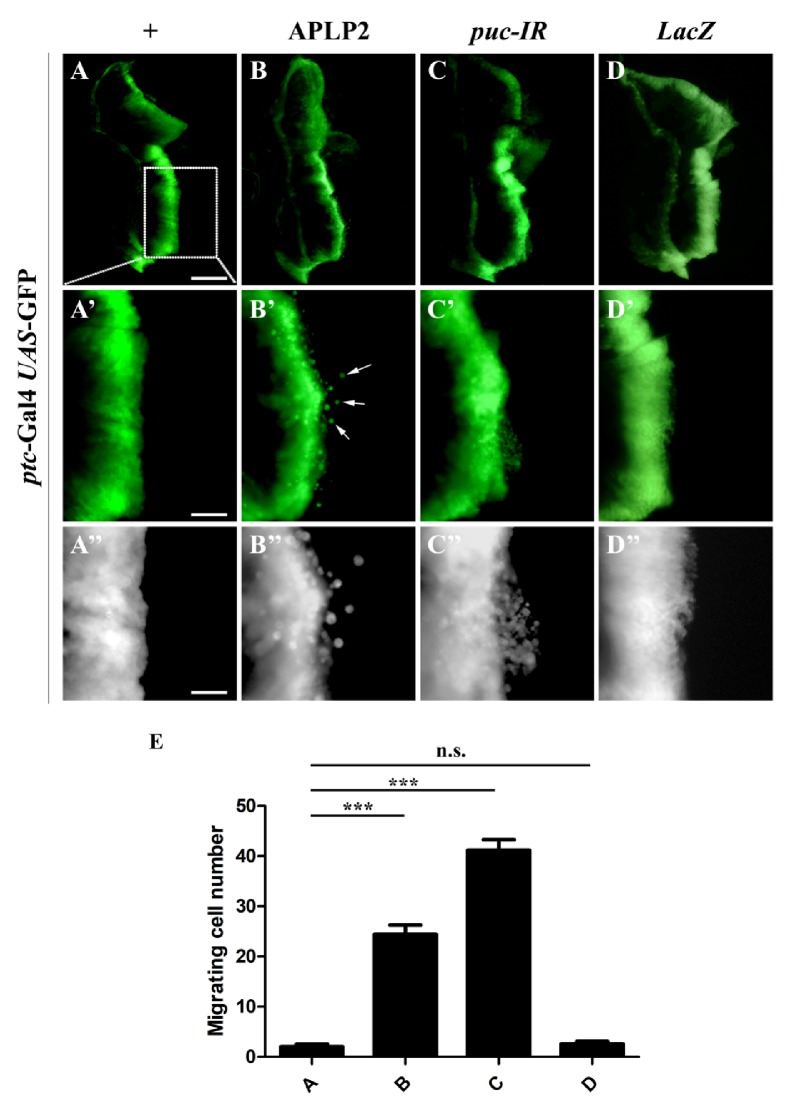
*APLP2 promotes cell migration in Drosophila*. Fluorescence micrographs of wing discs are shown. Compared with the* ptc*-Gal4* UAS*-GFP control (**A–A”**), expression of APLP2 induced mild cell migration behavior (**B–B”**). Activation of JNK signaling by depleting* puc* also triggered cell migration and served as a positive control (**C–C”**), while expression of* LacZ* served as a negative control (**D–D”**).** A'–D'** and** A”**–**D” **are high magnifications of** A–D**. (**E**) Quantification of the cell migration phenotypes, which were classified into four categories based on the number of GFP-labelled cells migrated to the posterior compartment. None: no migrated cells; Weak: 1-5 cells; Moderate: 6-20 cells; Strong: >20 cells. More than 20 discs were examined for each genotype. The crosses were performed at 29°C. *∗∗∗*, P<0.001; n.s., no significance. Scale bars in** A**,** A'**, and** A”** represent 200 *μ*m, 100 *μ*m, and 50 *μ*m, respectively. The genotypes used in the figure are as follows:* ptc*-Gal4* UAS*-GFP/+ (**A–A”**),* ptc*-Gal4* UAS*-GFP/*UAS*-APLP2 (**B–B”**),* ptc*-Gal4* UAS*-GFP/*UAS*-*puc-IR* (**C–C”**), and* ptc*-Gal4* UAS*-GFP/+;* UAS*-*LacZ*/+ (**D–D”**).

The expression level of APLP2 is increased in many tumor cells suggesting that APLP2 may play a vital role in tumor formation and metastasis[13, 33, 34]. To examine the exact function of APLP2 in cell migration* in vivo*, we ectopically expressed APLP2 along the anterior/posterior (A/P) compartment boundary in 3^rd^ instar larval wing discs, which has been commonly used to investigate the migrating phenotype in* vivo *[35]. We noticed that* patched*-Gal4 (**Figures 1(A)–1(A”)**) driven expression of APLP2 in the wing disc produced a dosage-dependent invasive phenotype with GFP-labelled cells diverted from the A/P boundary to the posterior part (**Figures 1(B)–1(B”), 1(E), Figure S1**), while expression of* LacZ* failed to induce such phenotypes (**Figures 1(D)–1(D”), and 1(E)**). The c-Jun N-Terminal Protein Kinase (JNK) signaling has been implicated in a wide range of cellular functions including cell death and migration [32, 36–40]. Consistently, RNAi-mediated depletion of* puckered *(*puc*), a negative regulator of JNK signaling [41, 42], promoted a cell migrating phenotype (**Figures 1(C)–1(C”), and 1(E)**). These data indicate that APLP2 is able to trigger cell migration* in vivo*, which phenocopies that of JNK activation.

### 3.2. JNK is Required for APLP2-Induced Cell Migration

**Figure 2:**
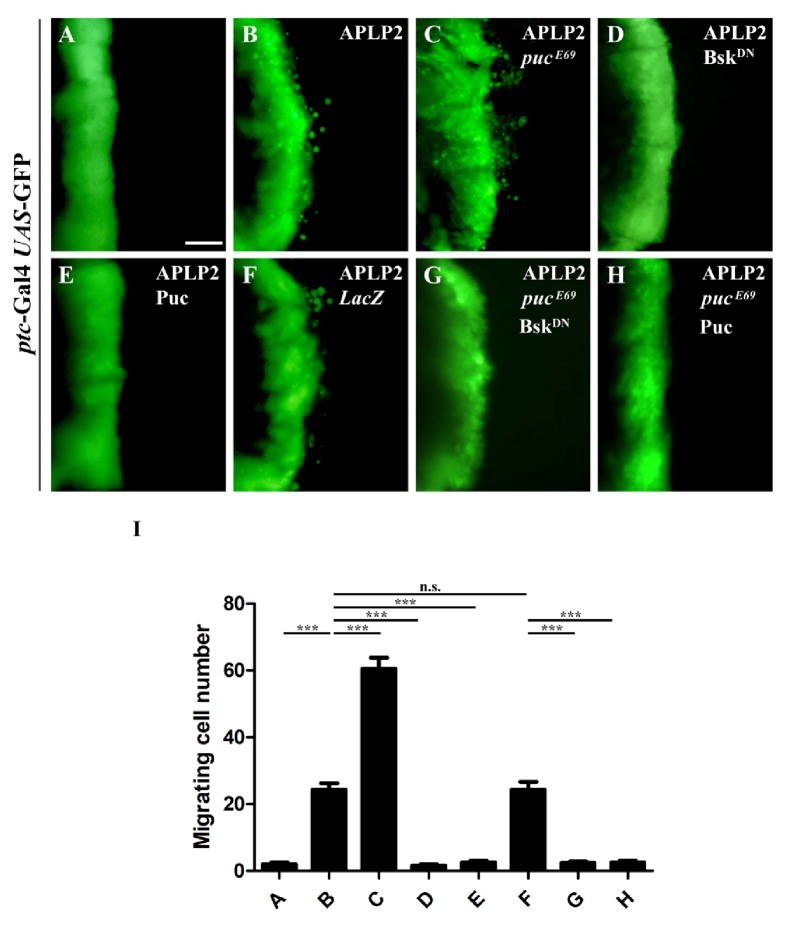
*APLP2 induces JNK-dependent cell migration*. Fluorescence micrographs of wing discs are shown. Compared with the* ptc*-Gal4* UAS*-GFP control (**A**), APLP2-induced cell migration (**B**) was exacerbated in heterozygous* puc* mutants (**C**) and suppressed by the expression of Bsk^DN^ (**D**) or puc (**E**) but remained unaffected by* LacZ* expression (**F**). The* puc* mutant-enhanced APLP2 cell migration phenotype was suppressed by the expression of Bsk^DN^ (**G**) or puc (**H**). (**I**) Quantification of the migration phenotypes in** A–H**. The crosses were performed at 29°C. *∗∗∗*, P <0.001. Scale bar in** A **represents 100 *μ*m. The genotypes used in the figure are as follows:* ptc*-Gal4* UAS*-GFP/+ (**A**),* ptc*-Gal4* UAS*-GFP/*UAS*-APLP2 (**B**),* ptc*-Gal4* UAS*-GFP/*UAS*-APLP2; *puc*^E69^/+ (**C**),* ptc*-Gal4* UAS*-GFP/*UAS*-APLP2;* UAS*-Bsk^DN^/+ (**D**),* ptc*-Gal4* UAS*-GFP/*UAS*-APLP2;* UAS*-puc/+ (**E**),* ptc*-Gal4* UAS*-GFP/*UAS*-APLP2;* UAS*-*LacZ*/+ (**F**),* ptc*-Gal4* UAS*-GFP/*UAS*-APLP2; *puc*^E69^/* UAS*-Bsk^DN^ (**G**),* ptc*-Gal4* UAS*-GFP/*UAS*-APLP2; *puc*^E69^/* UAS*-puc (**H**).

Since expression of APLP2 induced a migrating phenotype mimicking JNK activation in the wing disc, we hypothesized that JNK signaling pathway might be required for APLP2-triggered cell migration. To test this, we first elevated JNK signaling by deleting one copy of the endogenous* puc *gene encoding a JNK phosphatase that negatively regulates JNK activity [41, 42]. Compared with the* ptc-*Gal4 control (**Figures 2(A) and 2(I)**), we observed that APLP2-induced cell migration phenotype (**Figures 2(B) and 2(I)**) was dramatically enhanced in heterozygous* puc*^*E69*^ (**Figures 2(C) and 2(I)**) or* puc*^*H246*^ (**Figure S2**) mutants, while neither mutant alone could produce any migration phenotype [43], suggesting a genetic interaction between APLP2 and the JNK signaling in promoting cell migration. To further probe the role of JNK signaling in APLP2-induced cell migration, we blocked JNK pathway by expressing a dominant negative form of* Drosophila* JNK, Bsk [44], or the JNK phosphatase puc. We found that APLP2-induced cell migration was significantly suppressed by the expression of Bsk^DN^ or puc but remained unaffected by the expression of* LacZ *(**Figures 2(D)–2(F), and 2(I)**). Furthermore, blocking JNK signaling also inhibited APLP2-induced,* puc*^*E69*^-enhanced cell migration phenotype (**Figures 2(G), 2(H), and 2(I)**). Thus, we conclude that APLP2 induces JNK-dependent cell migration* in vivo*.

### 3.3. APLP2 Triggers JNK Activation* In Vivo*

**Figure 3:**
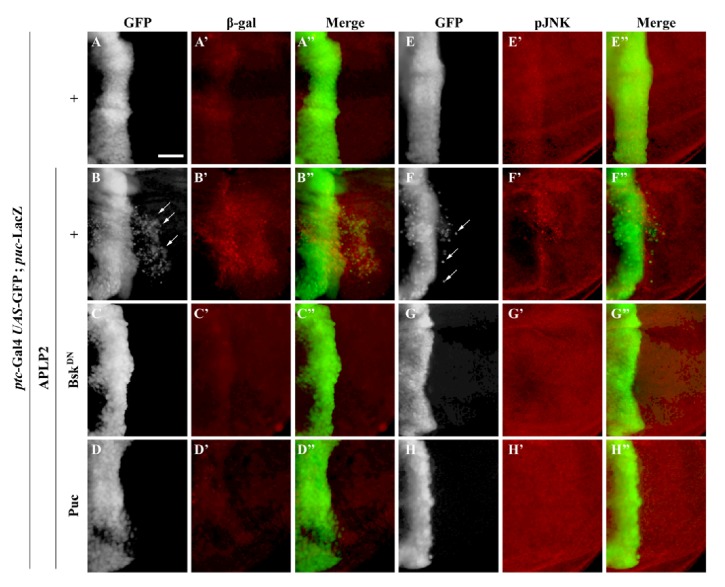
*APLP2 triggers JNK signaling activation*. Fluorescence micrographs of wing disc are shown. Compared with the* ptc*-Gal4* UAS*-GFP control (**A–A”**,** E–E”**), ectopic expression of APLP2 activated* puc-LacZ *expression (**B–B”**) and JNK phosphorylation (**F–F”**), which were impeded by the expression of Bsk^DN^ (**C–C”**,** G–G”**) or puc (**D–D”**,** H–H”**). The crosses were performed at 29°C. Scale bar in** A **represents 100 *μ*m. The genotypes used in the figure are as follows:* ptc*-Gal4* UAS*-GFP/+;* puc*-LacZ/+ (**A–A”**,** E–E”**),* ptc*-Gal4* UAS*-GFP/*UAS*-APLP2;* puc*-LacZ/*+* (**B–B”**,** F–F”**),* ptc*-Gal4* UAS*-GFP/*UAS*-APLP2;* puc*-LacZ/*UAS*-Bsk^DN^ (**C–C”**,** G–G”**),* ptc*-Gal4* UAS*-GFP/* UAS*-APLP2;* puc*-LacZ/*UAS*-puc (**D–D”**,** H–H”**).

The above data suggest that APLP2 promotes JNK-mediated cell migration* in vivo*. To investigate whether APLP2 is able to activate JNK signaling, we checked the expression of a* puc*-LacZ reporter, a commonly used readout of JNK signaling[27], and JNK phosphorylation in the wing disc. We found that APLP2 was sufficient to induce* puc*-LacZ expression (**Figures 3(B)–3(B”), Figure S3B**) and JNK phosphorylation (**Figures 3(F)–3(F”)**) in wing discs, compared with the* ptc*-Gal4 control (**Figures 3(A)–3(A”), 3(E)–3(E”)**). Consistent with the cell migration data, APLP2-induced* puc*-LacZ expression and JNK phosphorylation was considerably impeded by the expression of Bsk^DN^ (**Figures 3(C)–3(C”), 3(G)–3(G”), Figure S3C**) or puc (**Figures 3(D)–3(D”), 3(H)–3(H”)**). Collectively, the data suggest that APLP2 expression is sufficient to trigger JNK activation in the wing disc.

To investigate whether APLP2 could induce JNK activation in other tissues, we checked the salivary glands where* ptc*-Gal4 is also expressed. Compared to the control (**Figure S4A**), expression of APLP2 induced JNK signaling activation, as revealed by the* puc*-LacZ expression in the gland (**Figures S4B**). Expression of Bsk^DN^ fully suppressed both the endogenous and the ectopically activated expression of* puc*-LacZ (**Figure S4C**). Together, the data demonstrate that APLP2 is able to activate JNK signaling in a nontissue specific manner.

### 3.4. APLP2 Induces JNK-Mediated MMP1 Expression

**Figure 4:**
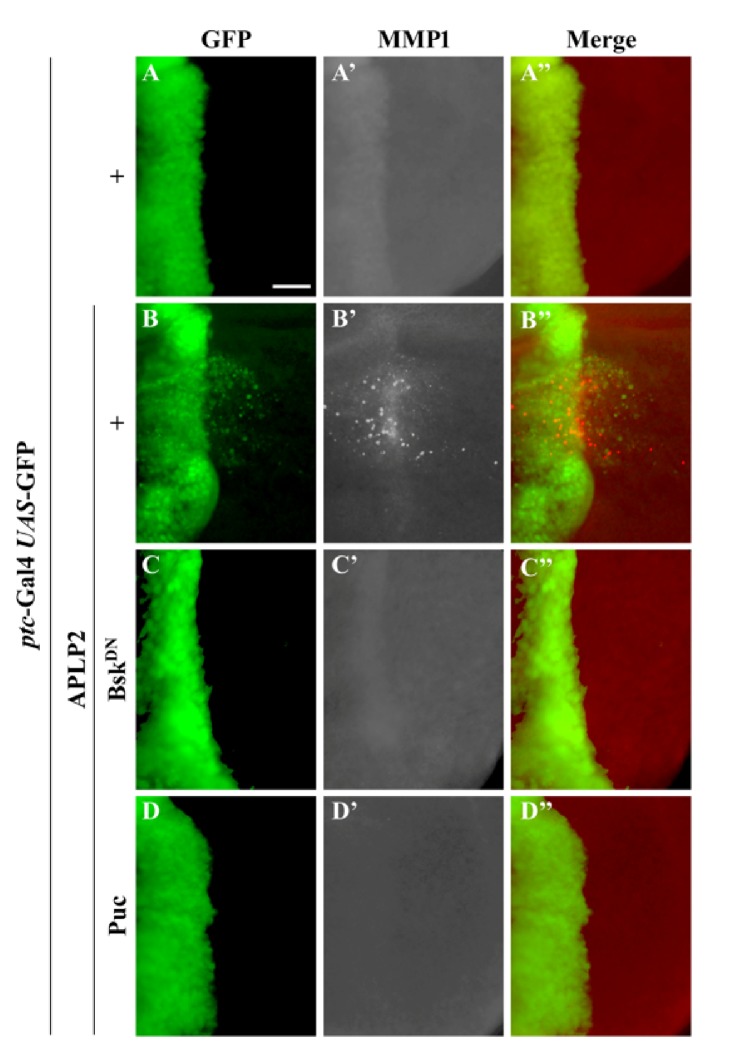
*APLP2 induces JNK-mediated MMP1 expression*. Fluorescence micrographs of wing discs are shown. Compared with the* ptc*-Gal4* UAS*-GFP control (**A–A”**), ectopic expression of APLP2 activated MMP1 expression (**B–B”**), which was blocked by expressing Bsk^DN^ (**C–C”**) or puc (**D–D”**). The crosses were performed at 29°C. Scale bar in** A **represents 100 *μ*m. The genotypes used in the figure are as follows:* ptc*-Gal4* UAS*-GFP/+;* puc*-LacZ/+ (**A–A”**),* ptc*-Gal4* UAS*-GFP/*UAS*-APLP2;* puc*-LacZ/*+* (**B–B”**),* ptc*-Gal4* UAS*-GFP/*UAS*-APLP2;* puc*-LacZ/*UAS*-Bsk^DN^ (**C–C”**),* ptc*-Gal4* UAS*-GFP/* UAS*-APLP2;* puc*-LacZ/*UAS*-puc (**D–D”**).

JNK-dependent cell migration is mediated by transcriptional upregulation of the matrix metalloproteinase MMP1[45, 46], which is required for the degradation of basement membrane and serves as a hall marker for cell migration behaviors in* Drosophila* [47–50]. Consistently, expression of APLP2 driven by* ptc*-Gal4 induced MMP1 expression in the wing disc (**Figures 4(B)–4(B”)**), which was dramatically suppressed by the expression of Bsk^DN^ (**Figures 4(C)–4(C”)**) or puc (**Figures 4(D)–4(D”)**). Intriguingly, APLP2 induced both autonomous and nonautonomous JNK phosphorylation (**Figures 3(F”)**) and MMP1 expression (**Figures 4(B”)**), which have been previously reported for other migration-promoting genes [37, 38]. Hence, APLP2 is able to induce JNK-mediated MMP1 activation, which is necessary for basement membrane degradation and cell migration. Similar results were observed in the P-compartment of wing discs when APLP2 expression was initiated by* engrailed*-Gal4 (*en*-Gal4) (**Figure S5**). Finally, we examined the role of MMP1 in APLP2-induced cell migration. We found that RNAi-mediated MMP1 depletion impeded APLP2-induced cell migration (**Figure S6**). Thus, ectopic expression of APLP2 is able to induce JNK-mediated MMP1 upregulation, which is crucial for basement membrane degradation and cell migration. Actin accumulation is a key hint for the cell migration phenotype [45, 46], and APLP2 was shown to modulate actin cytoskeleton in pancreatic cancer cells [13, 33, 34]. Consistently, we found that APLP2 expression could induce actin polymerization in the wing disc (**Figure S7**).

APLP2 expression is elevated in certain pancreatic and prostate cancer cells as well as in breast cancer samples, while downregulated in lymphoma cells and lung neuroendocrine tumors, implying a controversial role in tumor progression [13, 33, 34]. In this study, we investigated the* in vivo *function of APLP2 in* Drosophila* wing disc epithelia. Our data indicate that APLP2 is able to promote JNK-dependent cell migration* in vivo*. Mechanistically, APLP2 activates JNK signaling through the phosphorylation of JNK, which upregulates the expression of MMP1 that is essential for basement membranes degradation and cell migration. Our previous work showed that expression of APLP2 could induce the expression of apoptotic gene* hid* and apoptosis[51], yet APLP2-induced cell migration was not blocked by the expression of baculovirus* p35* (**Figure S8**), indicating APLP2-induced cell migration is independent of apoptosis. Consistent with our* in vivo* results, Chinese hamster ovary (CHO) cells overexpressing APLP2 exhibit increased chemotaxis toward type IV collagen and fibronectin [16], whereas depletion of APLP2 in pancreatic cancer cells resulted in reduced migration and invasion ability [13, 33, 34]. Intriguingly, comparable expression of APLP1[52] triggers stronger cell migration than APLP2 in Drosophila[43], suggesting both amyloid precursor-like proteins can promote cell migration* in vivo*, albeit at different efficiencies. Consistent with our finding, APLP1 and APLP2 are found to be increased in cancers [13, 33, 34] and knock-down of APLP2 in pancreatic cancer cells reduced the ability of cell migration[13].Yet it remains to be elucidated whether JNK signaling plays a crucial role in APLP2-induced cell migration and tumor invasion in mammals.

## Data Availability

No data were used to support this study.

## Supplementary Materials

Supplementary 1
* Figure S1: APLP2 induced dosage-dependent cell migration*. Fluorescence micrographs of wing discs are shown. Compared with the ptc-Gal4 UAS-GFP control** (A)**, APLP2-induced cell migration** (B)** was enhanced by adding another copy of APLP2** (C)**.** (D)** Quantification of migration phenotype in** A–C**. The crosses were performed at 29°C. *∗∗∗*, P <0.001. Scale bar in** A** represents 100 *μ*m. The genotypes used in the figure are as follows: ptc-Gal4 UAS-GFP/+** (A)**,* ptc*-Gal4* UAS*-GFP/UAS-APLP2** (B)**, and* ptc*-Gal4* UAS*-GFP* UAS*-APLP2 /*UAS*-APLP2** (C)**.

Supplementary 2
* Figure S2: loss of puc enhances APLP2-induced cell migration*. Fluorescence micrographs of wing discs are shown. Compared with the* ptc*-Gal4* UAS*-GFP control** (A)**, APLP2-induced cell migration** (B)** was aggravated in heterozygous* puc*^*H246*^ mutants** (C)**.** (D)** Quantification of migration phenotype in** A–C**. The crosses were performed at 29°C. *∗∗∗*, P <0.001. Scale bar in** A** represents 100 *μ*m. The genotypes used in the figure are as follows:* ptc*-Gal4* UAS*-GFP/+** (A)**,* ptc*-Gal4* UAS*-GFP/UAS-APLP2** (B)**, and* ptc*-Gal4* UAS*-GFP/*UAS*-APLP2; *puc*^H246^/+** (C)**.

Supplementary 3 
*Figure S3: APLP2 activates JNK signaling in the wing disc*. Light micrographs of wing discs are shown. The expression pattern of* ptc*-Gal4 was indicated by the red dashing line. Compared with the control** (A)**, expression of APLP2 induced* puc*-LacZ expression in the wing pouch** (B)**, which was blocked by expressing Bsk^DN^** (C)**. The crosses were performed at 25°C. Scale bar in** A **represents 200 *μ*m. The genotypes used in the figure are as follows:* ptc*-Gal4* UAS*-GFP/+;* puc*-LacZ/+** (A)**,* ptc*-Gal4* UAS*-GFP/*UAS*-APLP2;* puc*-LacZ/+** (B)**, and* ptc*-Gal4* UAS*-GFP/*UAS*-APLP2;* puc*-LacZ/*UAS*-Bsk^DN^** (C)**.

Supplementary 4 
*Figure S4: APLP2 activates JNK signaling in the salivary gland*. Light micrographs of salivary glands are shown. Compared with the control** (A)**, expression of APLP2 induced* puc*-LacZ expression in the salivary gland** (B)**, which was blocked by expressing Bsk^DN^** (C)**. The crosses were performed at 25°C. Scale bar in** A** represents 200 *μ*m. The genotypes used in the figure are as follows:* ptc*-Gal4* UAS*-GFP/+;* puc*-LacZ/+** (A)**,* ptc*-Gal4* UAS*-GFP/*UAS*-APLP2;* puc*-LacZ/+** (B)**, and* ptc*-Gal4* UAS*-GFP/*UAS*-APLP2;* puc*-LacZ/*UAS*-Bsk^DN^** (C)**.

Supplementary 5 
*Figure S5: APLP2 induces JNK-mediated MMP1 expression in the wing disc*. Fluorescence micrographs of wing discs are shown. Compared with the* en*-Gal4* UAS*-GFP control** (A–A”)**, ectopic expression of APLP2 in the posterior compartment of wing disc elevated MMP1 expression** (B–B”)**, which was blocked by expressing Bsk^DN^** (C–C”)**. The crosses were performed at 25°C. Scale bar in** A **represents 200 *μ*m. The genotypes used in the figure are as follows:* en*-Gal4* UAS*-GFP/+;** (A)**,* en*-Gal4* UAS*-GFP/*UAS*-APLP2** (B)**, and* en*-Gal4* UAS*-GFP/*UAS*-APLP2;* UAS*-Bsk^DN^/+**(C)**.

Supplementary 6 
*Figure S6: depletion of MMP1 compromises APLP2-induced cell migration*. Fluorescence micrographs of wing discs are shown. Compared with the* ptc*-Gal4* UAS*-GFP control** (A)**, APLP2-induced cell migration** (B)** was compromised by RNAi-mediated depletion of* mmp1 *** (C)**.** (D)** Quantification of the migration phenotypes in** A–C**. The crosses were performed at 29°C. *∗∗∗*, P <0.001, *∗∗*, P <0.01. Scale bar in** A **represents 100 *μ*m. The genotypes used in the figure are as follows:* ptc*-Gal4* UAS*-GFP/+** (A)**,* ptc*-Gal4* UAS*-GFP/*UAS*-APLP2** (B)**, and* ptc*-Gal4* UAS*-GFP/*UAS*-APLP2;* UAS-mmp1-IR*/+** (C)**.

Supplementary 7 
*Figure S7: APLP2 induces actin polymerization*. Fluorescence micrographs of wing discs are shown. Compared with the* ptc*-Gal4* UAS*-GFP control** (A–A”')**, APLP2 induces cell migration and actin remodeling** (B–B”')**. The crosses were performed at 29°C. Scale bar in** A **represents 100 *μ*m. The genotypes used in the figure are as follows:* ptc*-Gal4* UAS*-GFP/+** (A–A”')**, and* ptc*-Gal4* UAS*-GFP/*UAS*-APLP2; *puc*^E69^/+** (B–B”')**.

Supplementary 8 
*Figure S8: expression of p35 fails to block APLP2-induced cell migration*. Fluorescence micrographs of wing discs are shown. Compared with the* ptc*-Gal4* UAS*-GFP control** (A)**, APLP2-induced cell migration** (B)** cannot be blocked by expression of* p35 *** (C)**. The white arrow in** B** and** C** indicates the GFP-labelled migrating cells. The crosses were performed at 29°C. Scale bar in** A **represents 100 *μ*m. The genotypes used in the figure are as follows:* ptc*-Gal4* UAS*-GFP/+** (A)**,* ptc*-Gal4* UAS*-GFP/*UAS*-APLP2** (B)**, and* ptc*-Gal4* UAS*-GFP/*UAS*-APLP2;* p35*/+** (C)**.
