# Supplementary figures and images for: APLP2 Modulates JNK-Dependent Cell Migration in Drosophila
Source: Biomed Res Int. 2018 Jul 29;2018:7469714. doi: 10.1155/2018/7469714 (PMC6093063; doi:10.1155/2018/7469714)

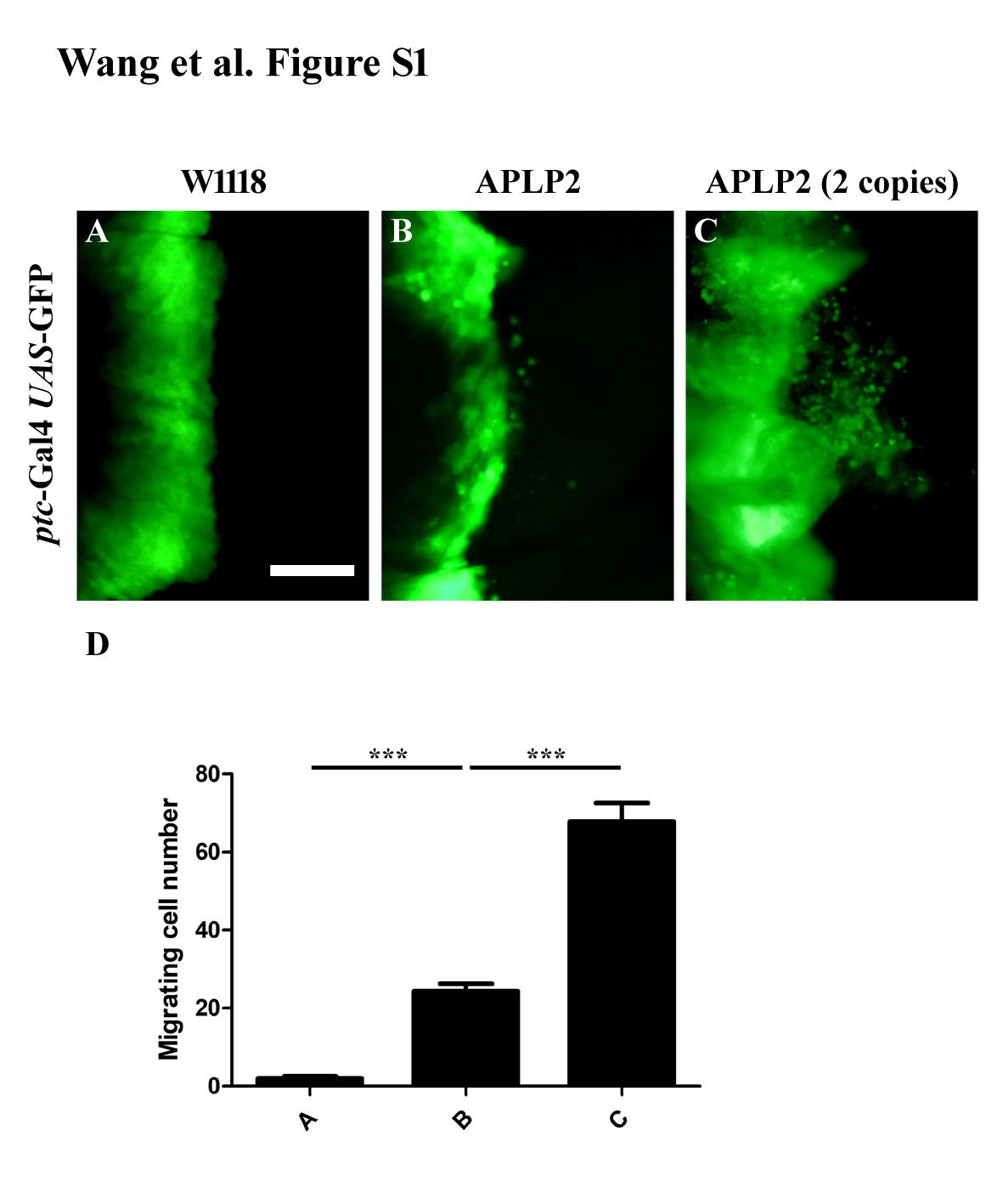

Supplement: Supplementary 1 — Figure S1: APLP2 induced dosage-dependent cell migration. Fluorescence micrographs of wing discs are shown. Compared with the ptc-Gal4 UAS-GFP control (A), APLP2-induced cell migration (B) was enhanced by adding another copy of APLP2 (C). (D) Quantification of migration phenotype in A–C. The crosses were performed at 29°C. ∗∗∗, P <0.001. Scale bar in A represents 100 μm. The genotypes used in the figure are as follows: ptc-Gal4 UAS-GFP/+ (A), ptc-Gal4 UAS-GFP/UAS-APLP2 (B), and ptc-Gal4 UAS-GFP UAS-APLP2 /UAS-APLP2 (C). [file 7469714.f1.docx]

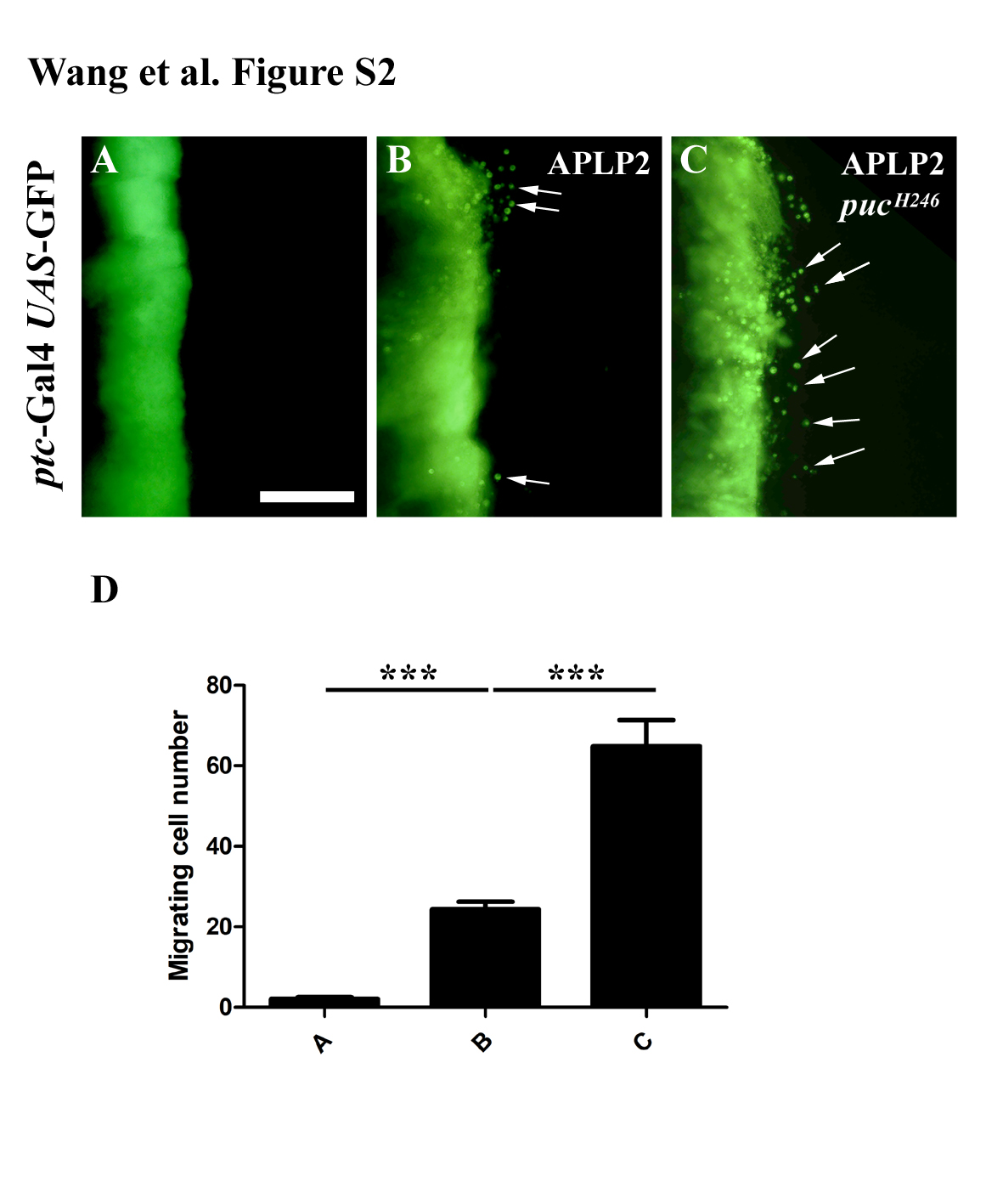

Supplement: Supplementary 2 — Figure S2: loss of puc enhances APLP2-induced cell migration. Fluorescence micrographs of wing discs are shown. Compared with the ptc-Gal4 UAS-GFP control (A), APLP2-induced cell migration (B) was aggravated in heterozygous pucH246 mutants (C). (D) Quantification of migration phenotype in A–C. The crosses were performed at 29°C. ∗∗∗, P <0.001. Scale bar in A represents 100 μm. The genotypes used in the figure are as follows: ptc-Gal4 UAS-GFP/+ (A), ptc-Gal4 UAS-GFP/UAS-APLP2 (B), and ptc-Gal4 UAS-GFP/UAS-APLP2; pucH246/+ (C). [file 7469714.f2.docx]

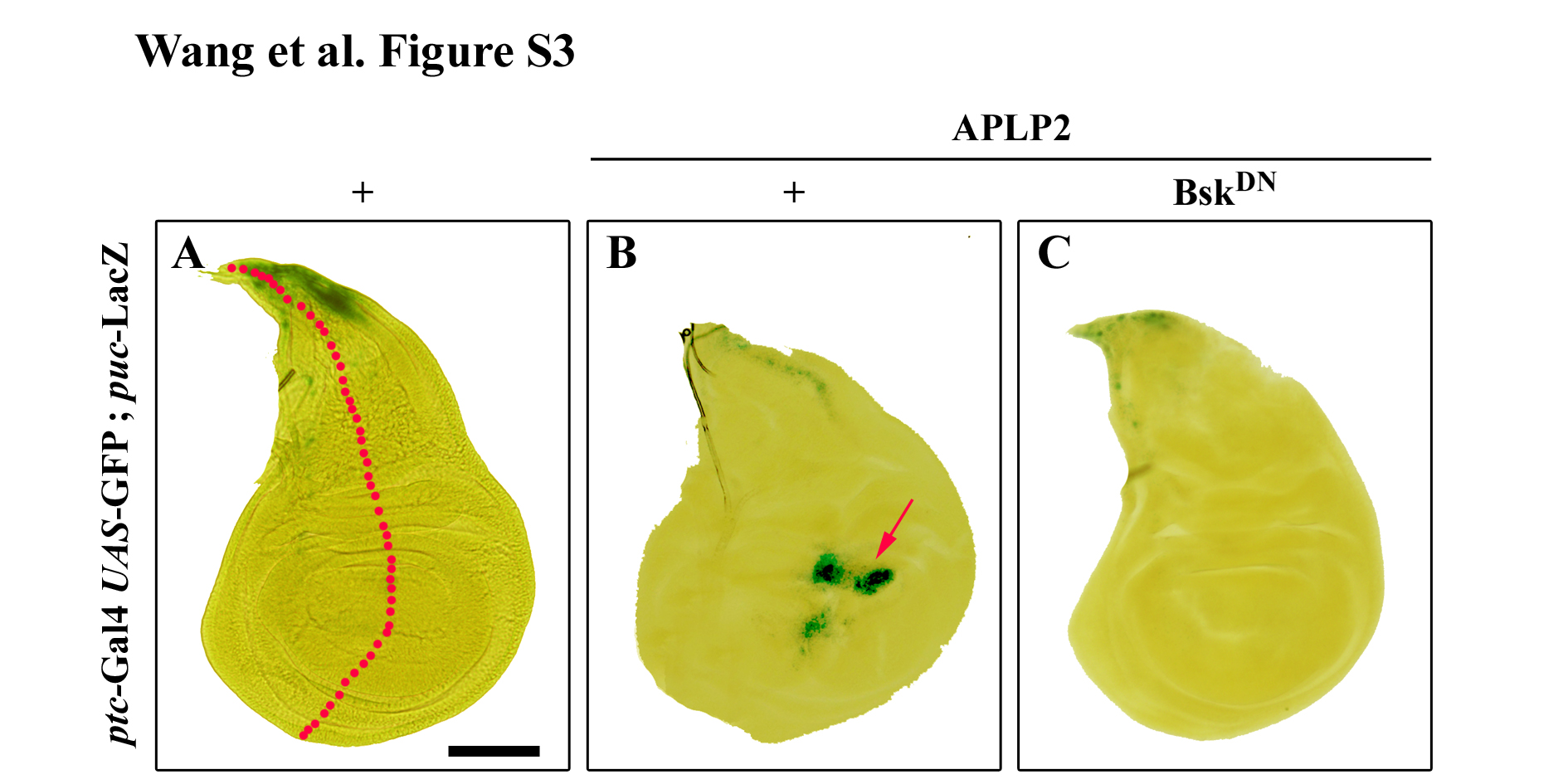

Supplement: Supplementary 3 — Figure S3: APLP2 activates JNK signaling in the wing disc. Light micrographs of wing discs are shown. The expression pattern of ptc-Gal4 was indicated by the red dashing line. Compared with the control (A), expression of APLP2 induced puc-LacZ expression in the wing pouch (B), which was blocked by expressing BskDN (C). The crosses were performed at 25°C. Scale bar in A represents 200 μm. The genotypes used in the figure are as follows: ptc-Gal4 UAS-GFP/+; puc-LacZ/+ (A), ptc-Gal4 UAS-GFP/UAS-APLP2; puc-LacZ/+ (B), and ptc-Gal4 UAS-GFP/UAS-APLP2; puc-LacZ/UAS-BskDN (C). [file 7469714.f3.docx]

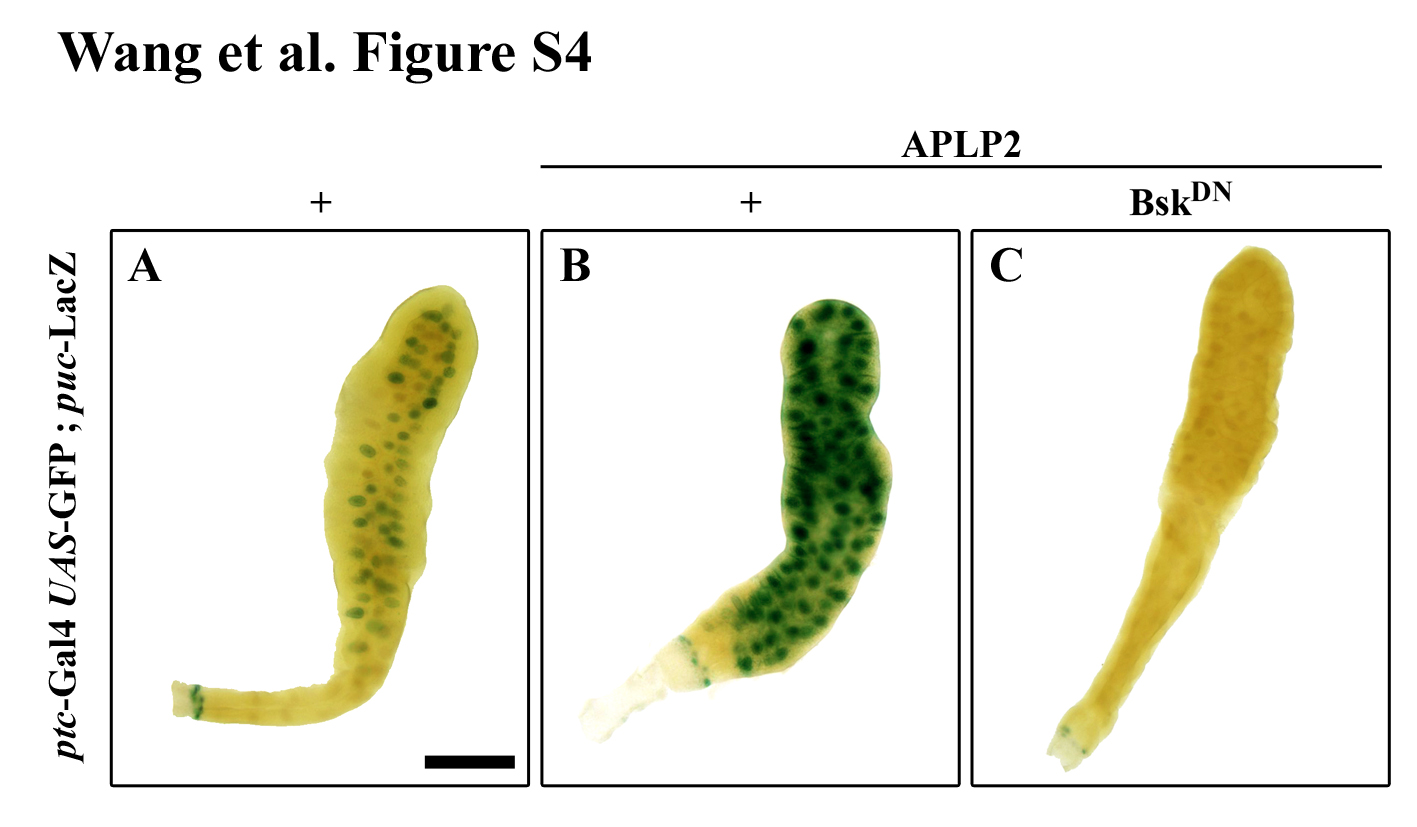

Supplement: Supplementary 4 — Figure S4: APLP2 activates JNK signaling in the salivary gland. Light micrographs of salivary glands are shown. Compared with the control (A), expression of APLP2 induced puc-LacZ expression in the salivary gland (B), which was blocked by expressing BskDN (C). The crosses were performed at 25°C. Scale bar in A represents 200 μm. The genotypes used in the figure are as follows: ptc-Gal4 UAS-GFP/+; puc-LacZ/+ (A), ptc-Gal4 UAS-GFP/UAS-APLP2; puc-LacZ/+ (B), and ptc-Gal4 UAS-GFP/UAS-APLP2; puc-LacZ/UAS-BskDN (C). [file 7469714.f4.docx]

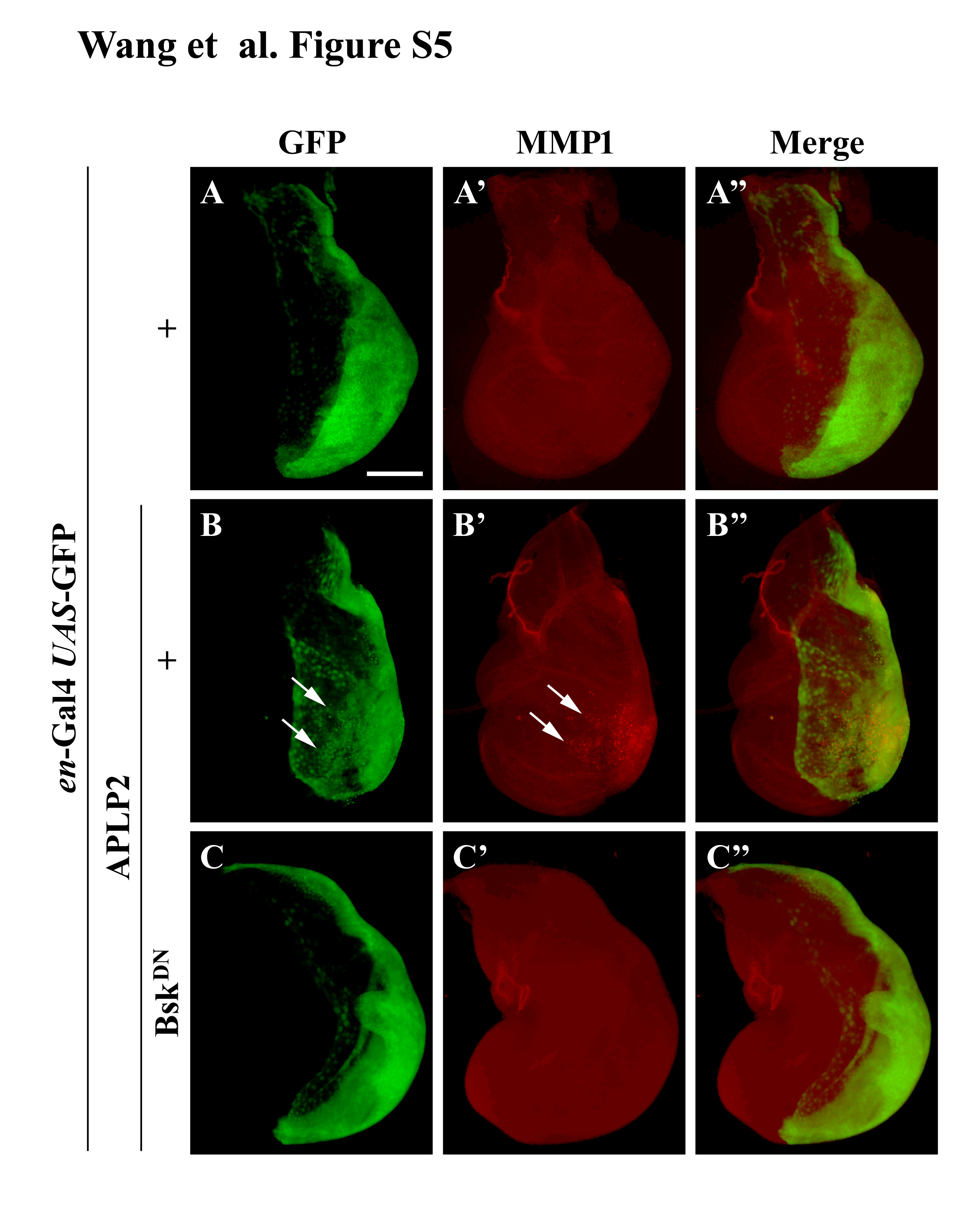

Supplement: Supplementary 5 — Figure S5: APLP2 induces JNK-mediated MMP1 expression in the wing disc. Fluorescence micrographs of wing discs are shown. Compared with the en-Gal4 UAS-GFP control (A–A”), ectopic expression of APLP2 in the posterior compartment of wing disc elevated MMP1 expression (B–B”), which was blocked by expressing BskDN (C–C”). The crosses were performed at 25°C. Scale bar in A represents 200 μm. The genotypes used in the figure are as follows: en-Gal4 UAS-GFP/+; (A), en-Gal4 UAS-GFP/UAS-APLP2 (B), and en-Gal4 UAS-GFP/UAS-APLP2; UAS-BskDN/+(C). [file 7469714.f5.docx]

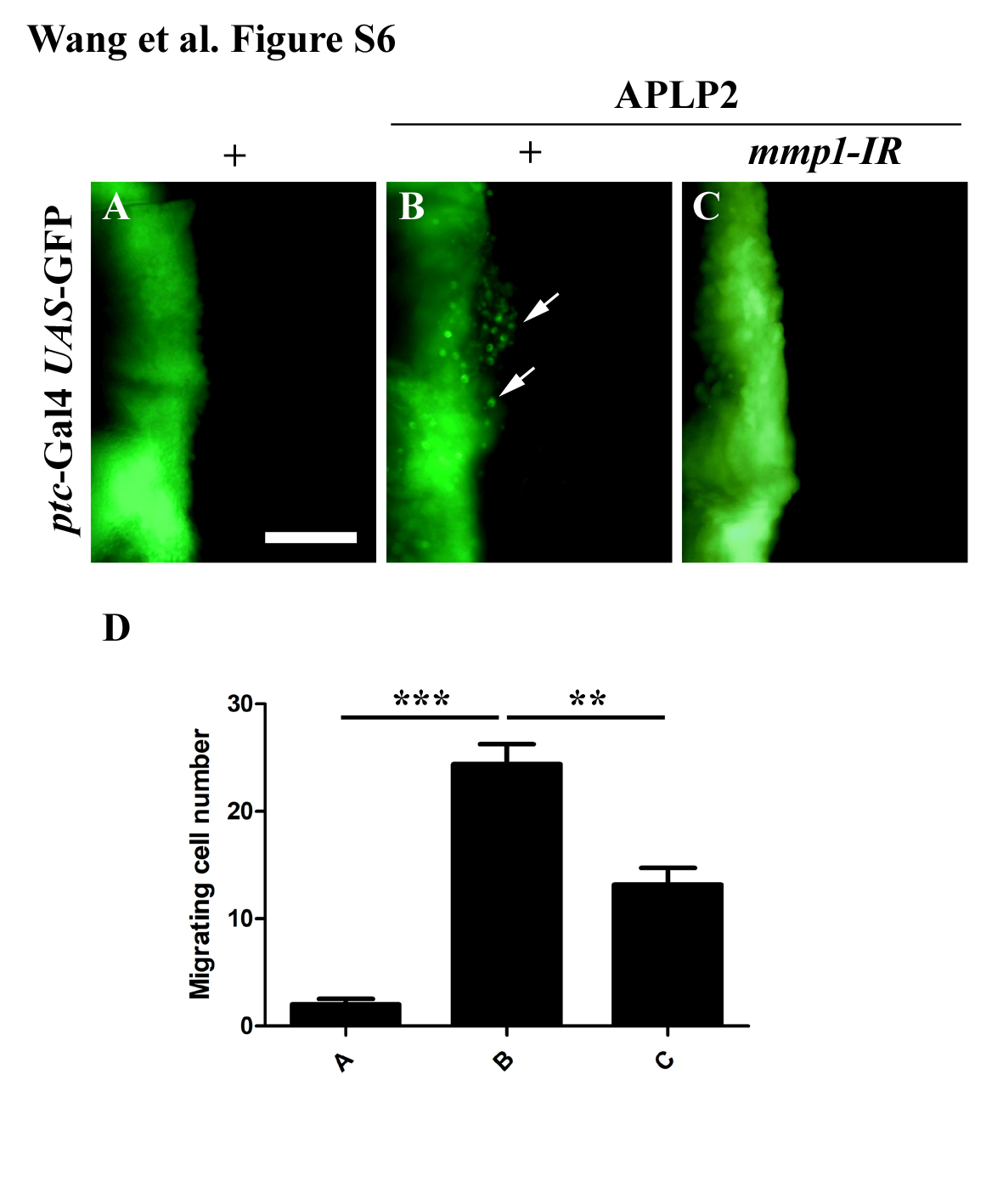

Supplement: Supplementary 6 — Figure S6: depletion of MMP1 compromises APLP2-induced cell migration. Fluorescence micrographs of wing discs are shown. Compared with the ptc-Gal4 UAS-GFP control (A), APLP2-induced cell migration (B) was compromised by RNAi-mediated depletion of mmp1 (C). (D) Quantification of the migration phenotypes in A–C. The crosses were performed at 29°C. ∗∗∗, P <0.001, ∗∗, P <0.01. Scale bar in A represents 100 μm. The genotypes used in the figure are as follows: ptc-Gal4 UAS-GFP/+ (A), ptc-Gal4 UAS-GFP/UAS-APLP2 (B), and ptc-Gal4 UAS-GFP/UAS-APLP2; UAS-mmp1-IR/+ (C). [file 7469714.f6.docx]

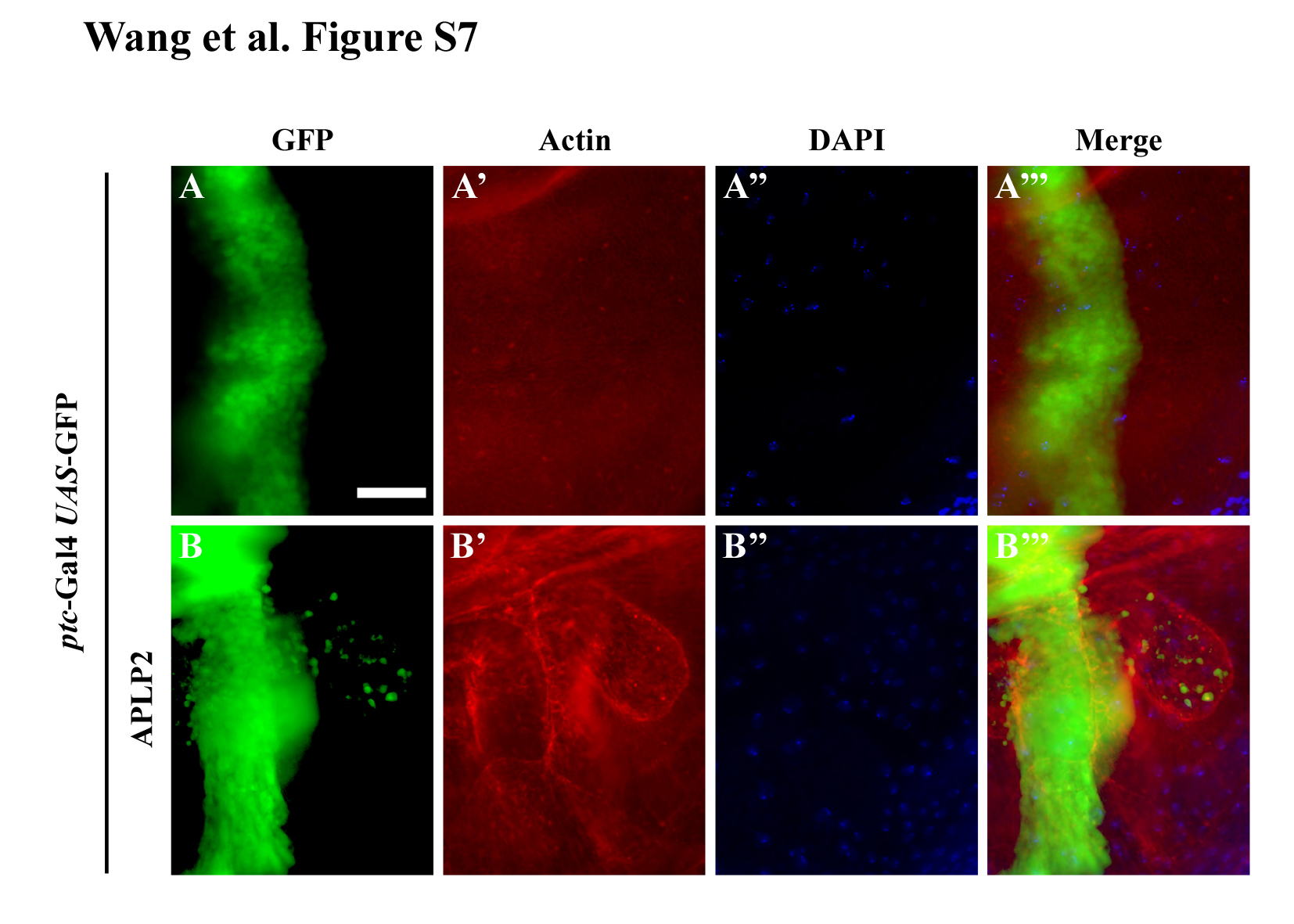

Supplement: Supplementary 7 — Figure S7: APLP2 induces actin polymerization. Fluorescence micrographs of wing discs are shown. Compared with the ptc-Gal4 UAS-GFP control (A–A”'), APLP2 induces cell migration and actin remodeling (B–B”'). The crosses were performed at 29°C. Scale bar in A represents 100 μm. The genotypes used in the figure are as follows: ptc-Gal4 UAS-GFP/+ (A–A”'), and ptc-Gal4 UAS-GFP/UAS-APLP2; pucE69/+ (B–B”'). [file 7469714.f7.docx]

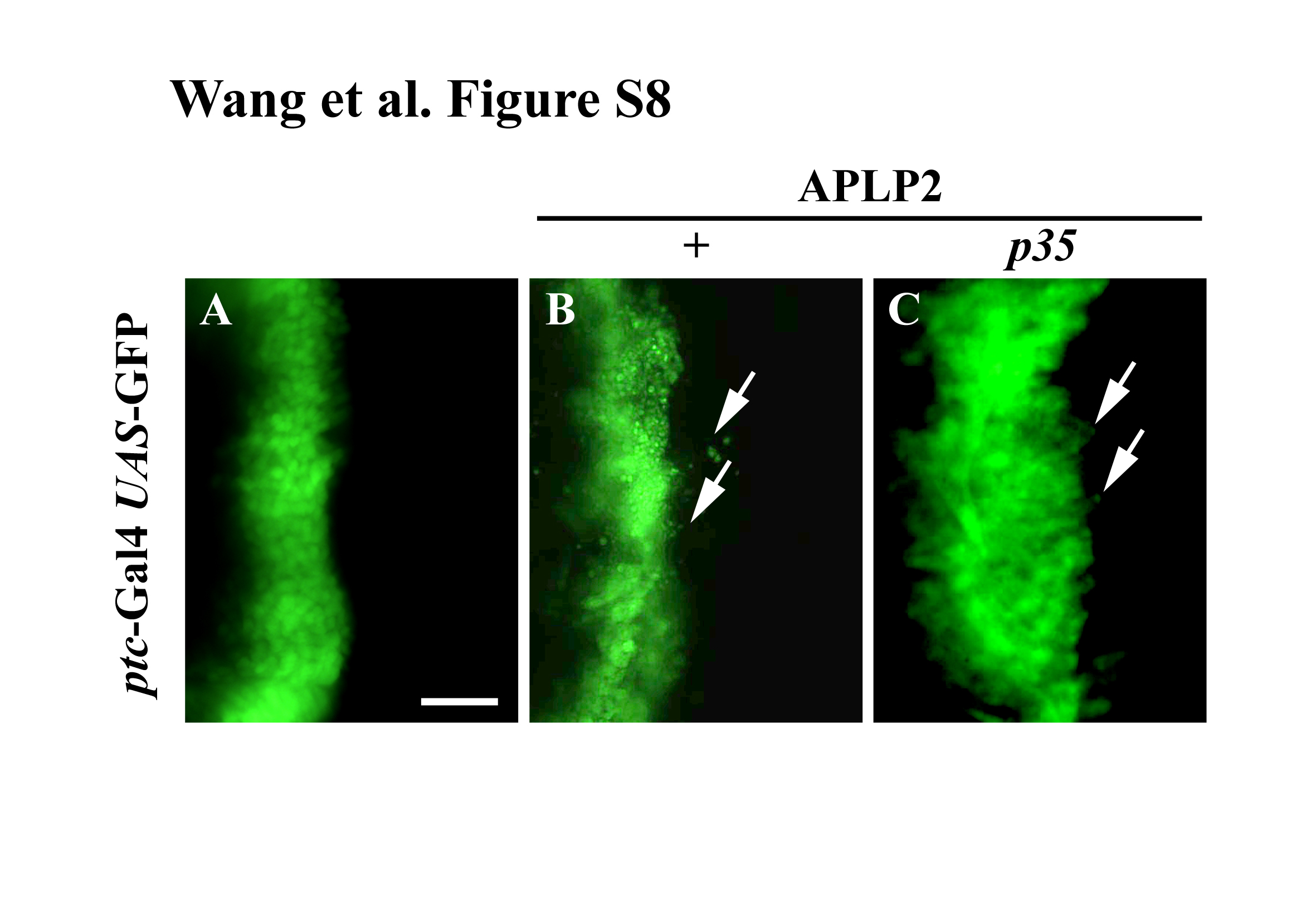

Supplement: Supplementary 8 — Figure S8: expression of p35 fails to block APLP2-induced cell migration. Fluorescence micrographs of wing discs are shown. Compared with the ptc-Gal4 UAS-GFP control (A), APLP2-induced cell migration (B) cannot be blocked by expression of p35 (C). The white arrow in B and C indicates the GFP-labelled migrating cells. The crosses were performed at 29°C. Scale bar in A represents 100 μm. The genotypes used in the figure are as follows: ptc-Gal4 UAS-GFP/+ (A), ptc-Gal4 UAS-GFP/UAS-APLP2 (B), and ptc-Gal4 UAS-GFP/UAS-APLP2; p35/+ (C). [file 7469714.f8.docx]
